# Management of Osteogenesis Imperfecta

**DOI:** 10.3389/fendo.2019.00924

**Published:** 2020-02-11

**Authors:** Stuart H. Ralston, Mark S. Gaston

**Affiliations:** ^1^Centre for Genetics and Experimental Medicine, MRC Institute of Genetics and Molecular Medicine, Western General Hospital, University of Edinburgh, Edinburgh, United Kingdom; ^2^Royal Hospital for Sick Children, Edinburgh, United Kingdom

**Keywords:** teriparatide (TPTD), clinical trials, fracture, bisphosphonates (BP), osteogenesis imperfecta (OI)

## Abstract

Osteogenesis imperfecta (OI) is the term used to describe a group of rare inherited skeletal disorders characterized by a greatly increased risk of fragility fractures (1). Mutations in several genes can cause OI but the condition is most commonly caused by mutations of *COLIA1* or *COL1A2* resulting in the production of collagen which is abnormal or present in reduced amounts. Fractures in OI are particularly common during childhood but the elevated fracture risk continues throughout life. Bone mineral density (BMD) can be reduced in OI but the magnitude of increase in fracture risk is far greater than can be accounted for by low BMD, highlighting that a key mechanism of bone fragility is reduced bone quality due to defects of bone matrix and mineralization. A multidisciplinary approach is needed to optimize management of OI, with input from physicians, orthopedic surgeons, physiotherapists, occupational therapists, and other allied health professionals. Orthopedic surgery plays a key role both in the fixation of fractures and in the correction of limb deformities. Bisphosphonates have been widely used in the treatment of children and adults with OI. Although there is good evidence that they increase BMD, it is uncertain to what extent they reduce fracture risk. Clinical trials of bone anabolic drugs such as teriparatide and inhibitors of sclerostin have also been studied; although they increase BMD, studies of these agents have not been powered to look at fracture endpoints. Various other treatment modalities including denosumab, and cell therapy have been explored but haven't gained acceptance in routine clinical practice. There have been huge advances in understanding the pathogenesis of OI but these have not been accompanied by advances in treatment. This signals need for well-designed clinical trials with fracture endpoints in OI, both with existing agents and with the newer therapeutic agents that are now starting to emerge.

## Introduction

Osteogenesis imperfecta is the term used to describe a group of inherited disorders characterized by multiple low trauma fractures, first presenting in infancy. Depending on the subtype, other features may be observed such as bone deformity, growth retardation, dental abnormalities, blue sclera, hearing loss, and ligament laxity.

The Sillence classification which was devised in 1979 (1), divided patients with OI into four subtypes based on clinical severity, ranging from mild to lethal. As new genes for osteogenesis imperfecta have been discovered a new classification system has been suggested (2) which introduces new subtypes related to the underlying genetic abnormality while retaining the Sillence classification for defects associated with mutations in the type 1 collagen genes.

The genetics of OI have recently been the subject of two recent comprehensive reviews (2, 3) and is also discussed in detail in another article in this series. In view of this, the gene mutations responsible for OI will be referred to only briefly in this article (Table 1). According to the new classification system, group A subtypes of OI are caused by defects in collagen synthesis, structure, or processing. The vast majority of patients with OI fall into this category. It has been estimated that between 85 and 90% of individuals with group A OI carry a mutation in *COLIA1 and COLIA2* which are the genes that encode the alpha 1 and alpha 2 chains of type 1 collagen. These are dominant mutations that impair the ability of type collagen to assemble normally or reduce the amount of collagen produced due to null mutations that evoke nonsense mediated RNA decay. Although, it was traditionally considered that null alleles were the underlying genetic defect in most individuals with Type I OI, recent information suggests that up to one third have a missense mutation in collagen (4). Mutations in the *BMP1* gene also cause a recessive form of OI that falls within group A classification since abnormalities of collagen assembly occur due to the protease function of *BMP1*. Group B subtypes of OI are recessively inherited and are due to mutations in genes that are responsible for post-translational modification of collagen. These are severe disorders which present in early infancy Group C subtypes can be dominantly or recessively inherited and most result in a moderate to severe phenotype. They are caused by mutations in genes that play a role in collagen folding and crosslinking of collagen. Heterozygous mutations in *P4HB* deserve special mention since that can cause a mild OI phenotype (5) as well as the Cole-Carpenter syndrome (6) in which an OI phenotype is accompanied by other features such as craniosynostosis, proptosis, hydrocephalus, and other distinctive facial features. Group D subtypes of OI are characterized by abnormalities of mineralization. Mutations in *IFTM5* cause an autosomal dominant form of OI where there is increased mineralization, whereas mutations in *SERPINF1* lead to a recessive form of OI characterized by reduced mineralization. Finally, group E subtypes of OI are recessively inherited severe forms of the disease which are caused by mutations in genes that affect osteoblast differentiation including *SP7, WNT1*, and *CREB3L1*.

**Table 1:** Clinical and genetic subtypes of osteogenesis imperfect.

**Group**	**Subtype**	**Genes**	**Inheritance**	**Mechanism**
Group A	Type I	*COL1A1 or COL1A2*	AD	Defects in collagen synthesis, structure, or processing
	Type II	*COL1A1 or COL1A2*	AD	
	Type III	*COL1A1 or COL1A2*	AD	
	Type IV	*COL1A1 or COL1A2*	AD	
	Type XIII	*BMP1*	AD	
Group B	Type VII	*CRTAP*	AR	Post-translational modification of collagen
	Type VIII	*LEPRE1*	AR	
	Type IX	*PPB*	AR	
	Type XIV	*TMEM38B*	AR	
Group C	X	*SERPINH1*	AR	Collagen folding or cross-linking
	XI	*FKBP10*	AR	
	–	*PLOD2*	AR
	–	*P4HB^§^*	AD
Group D	V	*IFTM5*	AD	Defects in bone mineralization
	VI	*SERPINF1*	AR	
Group E	XII	*SP7*	AR	Defects in osteoblast differentiation
	XV	*WNT1*	AR	
	XVI	*CREB3L1*	AR	

§ *Mutations in P4HB have been associated with a Cole-Carpenter syndrome in which an OI-like phenotype occurs as part of a wider syndrome but recent reports indicate that P4HB mutations can also result in a mild OI phenotype*.

There have been huge advances in understanding the molecular basis of OI but unfortunately this has not been accompanied by similar progress in terms of treatment. Furthermore, there is very little information on the relationship between the subtype of OI and the response to treatment. Hopefully the greater understanding of the molecular basis of OI that we now have will provide an avenue for the development of more effective targeted therapies in the future.

## Pathophysiology of Bone Fragility in Osteogenesis Imperfecta

Bone fragility is greatly increased in osteogenesis imperfecta. Reduced bone mass (7) and abnormalities of cortical thickness and trabecular architecture play a role (8) but these abnormalities are compounded by defects in bone matrix, which profoundly affect bone quality. There is also evidence that rates of bone turnover are abnormally increased particularly in types III and IV OI (8, 9) which may also contribute to bone fragility. Increases in TGF signaling have been implicated in the pathogenesis of increased bone remodeling in animal models of OI (10, 11) but the role of this cytokine in human OI has not yet been investigated. In another study, serum concentrations of DKK1 and RANKL were found to be elevated in a series of 18 children with OI (both untreated and after treatment with neridronate) leading the authors to conclude that these cytokines may play a role in causing increased bone resorption and reduced one formation in OI (12).

A puzzling feature of OI that still remains poorly understood is increased mineralization of bone. This was first described by Boyde et al. (13), but subsequently had been confirmed in most types of OI by various investigators (14–18). This has relevance to the pathogenesis of fractures since bone that is highly mineralized is also more brittle.

## Overall Management Strategy in Osteogenesis Imperfecta

This review will mainly focus on options for medical management, but it is important to point out that optimal management utilizes a multidisciplinary team (MDT) approach (19). There isn't a strong evidence base for this in the sense that the efficacy of individual components hasn't been tested in randomized clinical trials but clinical experience suggests that the MDT approach is important for the management of OI. Typically, the MDT is co-ordinated by a physician who usually will make the initial diagnosis of OI, decide upon the need for medical therapy and referral to other members of the MDT.

### Occupational Therapy

Occupational therapy plays a central role, particularly in individuals with more severe forms of OI to assess the patient and advise on devices and mobility aids to allow affected individuals to optimize function.

### Physiotherapy

The physiotherapist provides a key role in the care of patients with OI from when they are first diagnosed. They can assist parents of young OI babies and children with safe handling and positioning of the baby followed by setting realistic goals at reaching developmental milestones, dependent on the severity of the individual patient's condition. Probably the most important of these milestones is for the child to gain independent mobility whether this is with walking aids or ultimately using a wheelchair. The long-term input of the physiotherapist is key to optimizing the musculoskeletal health of children with OI and to help lead them into an independent adulthood. There is evidence from observational studies that supervised exercise programmes can positively influence muscle strength and physical ability (20, 21). Anecdotal evidence suggests that improving muscle strength can also have beneficial effects in stabilizing joints in people with hypermobility which is a common feature of OI. There has been a tendency for people with OI to be discouraged from participating in sports but there are many examples of individuals with OI reaching a high level in sporting activities. This in turn can have beneficial effects on well-being and quality of life.

### Orthotics

Orthotics may also have a role, particularly the ankle foot orthoses (AFO) which can control the position of the distal lower extremity and may have a role in preventing the worsening of foot deformity. Removable splints for the upper limb can be used to help ambulation for example in aiding the use of a self-propelled wheelchair. An individualized approach to the use of splints is required and an experienced orthotist can be invaluable for these patients.

### Other Health Care Professionals

The input of other health care professionals including clinical psychologists, speech and language therapists, dieticians, and social workers all play an important role in the multidisciplinary team (19). The orthopedic surgeon also plays a critical role in OI management, not only in the management of fractures, but also in performing prophylactic surgery to both prevent and correct deformities. The principles of orthopedic management of OI are discussed in more detail later in this review.

## Principles of Medical Management of Osteogenesis Imperfecta

The medical management of osteogenesis imperfecta is currently based on giving drugs that are used to treat osteoporosis, working on the assumption that medications which increase bone density and reduce bone turnover might favorably influence clinical outcome and reduce fracture risk. Other strategies such as stem cell therapy have been investigated but remain within the realm of research rather than routine clinical practice.

## Bisphosphonates in Osteogenesis Imperfecta

Bisphosphonates are the most widely used agents for the treatment of osteogenesis imperfecta. In the present review, I will review the results that have been reported with individual bisphosphonates followed by an overall summary of the effects of bisphosphonates at the end of this section.

### Pamidronate

Pamidronate became widely used in children with OI following the observational study of Glorieux et al. (22) who treated 30 children with severe OI with intermittent infusions of pamidronate over a period of between 1 and 5 years using a dosage regimen of 1.5–3.0 mg/kg on 3 consecutive days at 4–6 monthly intervals. Most of the individuals had a severe phenotype such that about one-third had type III, and the remainder type IV with a few individuals that had clinical features of type V disease.

Following treatment, the authors reported that the most striking difference was an improvement in bone pain which was associated with an improvement in mobility. Bone mineral density assessed by DEXA also increased and the fracture rate decreased from 3.2 fractures per patient per year to 0.6 fractures per patient per year with time – a reduction of about 75%. The authors also noted an increase in vertebral area following treatment. The most common adverse effects were fever and a transient increase in bone pain which are well-recognized to occur with pamidronate in other indications (23). Reductions in serum calcium were also noted but these were usually asymptomatic. Since this wasn't a placebo-controlled study, further research was conducted to try and determine to what extent the reduction in fracture risk and increase in bone density was due to the therapeutic intervention or to skeletal growth or other confounding factors. To try and address this, Rauch and colleagues compared the increases in BMD following pamidronate therapy in 56 children with OI as compared with 167 patients who had not been treated. Regression analysis showed that pamidronate treated individuals had a greater increase in BMD than untreated subjects and this was further supported by a subgroup analysis of 51 treated individuals who were matched with 51 untreated individuals of a similar age and OI type (24). Another study of similar design was conducted by Land and colleagues who compared changes in vertebral shape in pamidronate treated individuals compared with controls matched for age and type of OI. This also showed that pamidronate seemed to preserve vertebral shape (25).

At the time of writing this review, a search of PubMed identified over 150 publications referring to the use of pamidronate in OI. These studies generally supported the findings noted by Glorieux in showing increased BMD, reduced fracture incidence and symptomatic improvement after treatment as compared with before but with one exception which compared pamidronate with alendronate (26) these were observational studies with no control group. While the data suggest that pamidronate increases BMD and may affect vertebral modeling it remains less clear to what extent the reduction in fracture rate is due to pamidronate or confounding factors such as the tendency for fracture rates to decrease spontaneously with skeletal growth in children with OI, even in the absence of treatment (27, 28).

### Alendronate

There have been three randomized trials of alendronate in OI, two in children and one in adults. DiMeglio and Peacock conducted a small randomized open study of 18 children with OI who were treated with oral alendronate 1 mg/kg/day or intravenous pamidronate 4 mg/kg every 4 months over a 2-year period (26). Bone density increased at the spine and total body to a similar extent with both treatments, but with a better response in type I as compared with type III and IV irrespective of the treatment given. The authors noted a reduction in fracture incidence during treatment with both drugs as had been reported in uncontrolled studies with pamidronate. Ward and colleagues conducted a double-blind randomized study of oral alendronate (n-109) and placebo (*n* = 30) in children with OI (29). The dose of alendronate was 5 mg daily in those <40 kg and 10 mg daily in those more than 40 kg. There was a significantly greater increase in BMD at the spine in the alendronate group as compared with placebo and this was accompanied by a significantly greater reduction in urinary N-telopeptide of type I collagen (uNTX/Cr) than in the placebo group. There was no significant difference in the proportion of patients with new fractures between the placebo group (92%) and alendronate group (83%), although the authors commented that the study had been powered to look at changes in BMD and not fracture. No difference between the groups was observed in secondary outcomes such as pain, mobility and physical activity. The alendronate was well-tolerated with an adverse event profile similar to that of placebo. Chevrel et al. (30) conducted a randomized double-blind trial or oral alendronate 10 mg daily vs. a matching placebo in 64 adults with OI over a 3-year period. Their average age was about 36 years and most had type I OI. All individuals received calcium and vitamin D supplements. There was a significant increase in hip BMD and spine BMD in the alendronate treated patients and this was accompanied by a decrease in biochemical markers of bone turnover. The fracture rate was similar in both groups; 11/31 (35%) in the placebo group experienced a non-vertebral fracture compared with 10/31 (32%) in the alendronate group. Two vertebral fractures occurred in the placebo group compared with none in the alendronate group and overall, 17 fractures occurred in each group during the treatment period. With regard to adverse events, pain score increased from baseline in the alendronate group in the intention to treat analysis but was not different in the per-protocol analysis. Gastro-intestinal adverse events were more common in the alendronate group occurring in 18/31 (58%) individuals compared with 5/31 (16%) in the placebo group.

### Risedronate

The effects of risedronate have been studied in three randomized trials of children with OI.

A dose-ranging study by Bishop et al. (31) compared the effects of risedronate in doses of 0.2, 1, and 2 mg/kg in 53 children with OI. The average age at entry to the study was 11 years and most of the participants had type I OI. The study was powered to show a 75% reduction in fracture rate, based on the pamidronate study of Glorieux et al. (22). No effect of either 1 or 2 mg/kg was observed on fracture incidence as compared with the 0.2 mg/kg dose but BMD increased significantly with the 2 mg/kg dose as compared with the lowest dose. No differences in functional outcomes such as pain and grip strength were found between the treatment groups. The authors referred to a dose-related effect of treatment on bowing deformities in the text of the paper but no data on bowing deformities were presented. A further study by Bishop randomized 147 children with OI to receive risedronate 2.5 or 5 mg daily (depending on body weight) or placebo for a period of 1 year, at which point all patients were switched to risedronate. The average at entry to the study was about 8 years and most participants had mild OI. The study was powered to detect a difference in BMD of 5% between the risedronate and placebo groups at 1 year. At the end of the placebo-controlled phase, BMD increased significantly in risedronate treated patients. As expected, urinary NTx and bone specific alkaline phosphatase values were reduced in the risedronate treated patients during the blinded phase of the study. The proportion of patients with new clinical fractures was lower in the risedronate group 29/94 (30.8%) vs. 24/49 (48.9%) of the placebo group. The difference between groups was significant (*p* = 0.0446). A time to event analysis also showed that the hazard ratio for fractures was 0.53 (95% CI, 0.31–0.92) for risedronate compared with placebo. There was a trend for fewer vertebral deformities in the risedronate group but the difference from placebo was not significant. A third study Rauch randomized 26 children with type I OI to receive risedronate or placebo (32). The dose of risedronate was 15 mg weekly for those with a body weight of <40 kg and 30 mg weekly for those >40 kg. Treatment was continued for 2 years. In the risedronate group, BMD increased significantly and serum NTX decreased by about 30%. The authors noted that the decrease in NTX was proportionately less than that observed in previous observational studies with pamidronate but the age of patients and severity of OI was different in these studies, making a direct comparison difficult (22). There was no significant difference between the risedronate and placebo groups in vertebral morphometry, grip strength or bone pain. Eleven fractures occurred in each group, a difference that was not significant. Adverse events were similar in the treatment groups.

### Neridronate

Neridonate is a nitrogen containing bisphosphonate that is structurally similar to pamidronate and alendronate but with six carbon atoms in the side chain as opposed to three with pamidronate and four with alendronate. It is currently licensed in Italy for the treatment of bone disease. Intravenous neridronate has been studied in one randomized controlled trial of children with OI performed by Gatti et al. (33). The trial was of 3 years duration but only the first year was placebo controlled at which point all patients were switched to neridronate which was given in a dose of 2 mg/Kg body weight by intravenous infusion every 3 months. Vitamin D supplements were also given to both groups in those with a vitamin D level of <50 nmol/l. The average age of participants was about 9 years and the majority had type I OI. During the placebo-controlled phase, BMD increased at the spine and hip to a significantly greater degree in the neridronate group. Gain of height and bone area in the lumbar spine were also reported to be greater during the first year in the neridronate group. During the placebo-controlled phase, 10/22 (45%) of the placebo group and 12/42 (28%) of the neridronate group had fractures; a difference that was not significant (OR 0.6, 95% CI 0.21–1.59). The number of fractures were fewer in the neridronate group however (OR 0.36, 95% CI 0.15–0.87, *p* < 0.05) and a similar reduction in fracture numbers was reported when the data were adjusted for baseline BMD, type of OI, and gender. However, the authors did not adjust for the proportion of individuals who had experienced fractures in the 12 months before the study, and this was higher in the placebo group (82 vs. 57%). In the open (observational) phase of the study, fracture rate decreased in both groups as expected. Another open study (34) randomized 46 adults with OI to receive neridronate 100 mg intravenously every 4 months or no treatment in a 2:1 ratio. The average age of participants 35 years and most had type I OI. There was a greater increase in spine and hip BMD in the neridronate group as compared with the untreated controls. Biochemical markers of bone turnover including bone specific ALP, serum CTX and urinary deoxypyridinoline/creatinine fell in both groups although the reductions tended to be greater in the neridronate group. Reporting of fracture data in this study was incomplete. The authors reported that 2 clinical fractures occurred in the placebo group during year 1 compared with 1 fracture in the neridronate group. No information was provided on fractures that occurred during the second year in the two groups. Flu-like symptoms were observed in 13/31 (42%) patients treated with neridronate but no serious adverse events were reported.

### Olpadronate

Olpadronate is a bisphosphonate which is structurally similar to pamidronate but with an additional methyl group on the nitrogen atom. There has been one randomized study with olpadronate in OI and that was performed by Sakkers and colleagues (35) who randomized 34 children with OI to receive olpadronate 10 mg daily (*n* = 16) or placebo (*n* = 18). The average age of participants at baseline was about 10 years. There was a mix of OI types in each group; about half of the individuals in the placebo group had type I OI compared with one third in the olpadronate group. Spine BMD increased in the olpadronate group as compared with placebo, but there was no difference between groups in grip strength, mobility and biochemical markers of bone turnover. Fractures were recorded in 12/18 (77%) of the olpadronate group compared with 8/16 (50%) of the placebo group; a difference that was not significant. The total number of fractures was greater in the placebo group however (50 vs. 18) and this was significant with a reported hazard ratio of 0.69 (95% CI 0.52–0.91). A subsequent analysis of quality of life in participants of the same study (36) revealed very few differences between groups with the exception of a marginal improvement in pain at one time point only.

### Summary

Randomized controlled trials of bisphosphonates in OI have consistently shown that BMD is increased and biochemical markers of bone turnover are decreased as compared with no treatment or placebo. There haven't been clear benefits in quality of life and functional status in controlled studies suggesting that the improvements in these domains that were seen in observational studies may not have been fully been attributable to the bisphosphate therapy. The data on fracture are also conflicting. A Cochrane review and a meta-analysis of randomized trials have both concluded that the effects of bisphosphonates on fracture rate are uncertain (27, 37), while also acknowledging that the studies performed so far have not generally been powered to detect a reduction in fracture incidence. A possible reason for the slightly disappointing results with bisphosphonates in terms of fracture reduction is that they increase mineralization of bone (38, 39) which might cause the bone to be more brittle.

## Denosumab

Denosumab is a monoclonal antibody directed against Receptor Activator of Nuclear Factor Kappa B ligand (RANKL) a molecule that plays an essential role in osteoclast activation. Through this action, denosumab acts as a highly potent inhibitor of osteoclastic bone resorption. When given in a dose of 60 mg subcutaneously every 6 months, denosumab has been shown to significantly reduce the risk of fractures in postmenopausal osteoporosis (40).

There have been some reports of denosumab use in OI, but no randomized trials. The first published case series of denosumab treatment in OI was by Hoyer-Kuhn et al. (41) who administered denosumab in a dose of 1 mg/kg every 12 weeks 6 months to 10 children with OI over a 4-year period. All individuals had previously been treated with bisphosphonates but these were discontinued 6 months before entry to the study. Calcium and vitamin D supplements were given routinely. The average age of participants was 7 years and most had type 1 OI. Bone density increased during the study but there were no changes in spine morphometry or general mobility. Urinary Deoxypyridinoline/Creatinine values (a marker of bone resorption) fell significantly after the first administration of denosumab with a nadir at day 8 and the values rose once again such that by 10 weeks, the values were approaching pre-treatment levels. Various adverse events were reported including one instance of hypocalcaemia.

Another study by the same group reported outcomes in a case series of four individuals with type VI OI treated with denosumab (42). Bone density and vertebral area increased with time in these individuals. Urinary DpD values decreased on day 14 after the injections and then tended to recover by day 28. In another study Trejo and colleagues reported outcomes in four individuals with type VI OI treated with denosumab. Somewhat surprisingly they reported the occurrence of hypercalcaemia and hypercalciuria following treatment. In addition, rapid bone loss at the lumbar spine was noted when the interval between injections was increased to 6 months (43). A systematic review of denosumab in children with OI by Li et al. (44) identified and evaluated the three studies above and concluded that further research was necessary to evaluate its role in treatment.

In summary there are limited data on the use of denosumab in OI and its effects on clinical outcome of the disease remains unclear.

## Bone Anabolic Agents in Osteogenesis Imperfecta

### Teriparatide

Teriparatide (TPTD) is the 1–34 fragment of parathyroid hormone. It has been successfully used for many years in the treatment of osteoporosis and has been found to be superior to bisphosphonates in the prevention of vertebral fractures in severe osteoporosis in observational studies (45). Moreover, TPTD was found to be superior to oral risedronate in preventing vertebral fractures in a double-blind randomized trial of individuals with severe osteoporosis (46). There has been one double blind randomized trial of Teriparatide in OI (47); in this study; Orwoll and colleagues randomized 79 adults with OI to receive TPTD 20 mcg daily or a matching placebo for an 18-month period. The average age of participants was 41 years and most had type I OI. There was a significantly greater increase in BMD in the TPTD group compared with placebo and biochemical markers of bone turnover (PINP and NTX) were both significantly elevated following TPTD therapy. The authors noted that changes in BMD and biochemical markers in response to TPTD were greater in those with type I OI as compared with those that had type III/IV. Fractures were observed in 11 (29%) of the TPTD group and 14 (36%) of the placebo group, a difference that was not significant (odds ratio 0.73, 95% CI 0.28–1.90). An observational study by Gatti and colleagues (48) treated 13 adults with type I OI with TPTD over an 18-month period. These individuals were selected for treatment on the basis that they had suffered fractures despite neridronate treatment. The study showed increased in BMD and biochemical markers with TPTD treatment. None of the patients experienced a fracture during TPTD therapy.

### Sclerostin Inhibitors

Romosozumab, a monoclonal antibody which neutralizes sclerostin. Through these effects, romosozumab inhibits bone resorption, probably by increasing production of osteoprotegerin by osteoblasts (49) and stimulates bone formation by counteracting the inhibitory effects of sclerostin on osteoblast activity (50). Interestingly, the bone forming effect of romosozumab is transient and is lost after about 12 months, even with continued treatment. Despite this, romosozumab is a powerful anabolic agent which has been found to be effective at increasing BMD and reducing the risk of fractures in postmenopausal osteoporosis when compared with placebo (51). Prompted by these observations, and preclinical studies which have suggested that sclerostin antibodies can increase bone mass and bone strength in several mouse models of OI (52), sestrusumab (BPS-804) a monoclonal antibody which neutralizes sclerostin is now being investigated in the treatment of adults with OI.

In a randomized phase 2a trial, Glorieux conducted a dose ranging study with sestrusumab in nine adults with osteogenesis imperfecta in comparison with a reference group who were randomly allocated to receive no treatment. Participants were predominantly male, with an average age of 30 years and similar numbers of individuals with type I, III, and IV OI were included in the study. A sequential design was used in the active group such that participants received an initial intravenous infusion of sestrusumab 5 mg/kg followed by a further infusion of 10 mg/kg after an interval of 2 weeks later and a final infusion of 20 mg/kg after a further interval of 2 weeks with a final study visit follow-up 21 weeks after the initial dose. The control group were followed up without treatment for 21 weeks. The primary efficacy endpoints were changes in biochemical markers of bone turnover but bone density was also measured by DEXA. Biochemical markers of bone formation including PINP and BSAP increased significantly in the active group when compared with the control group. Lumbar spine BMD also increased in the active group but did not change significantly in the control group. The treatment was generally well-tolerated. The authors concluded that BPS-804 was effective at stimulating bone formation and bone density in OI. A further phase 2b study is now in progress with four different doses of sestrusumab in 100 adults with OI (Asteroid study, NCT03118570) The study has completed recruitment and is currently in follow-up.

### TGF Beta Inhibition

There is evidence that TGFβ signaling is increased two mouse models of OI (10); one with targeted inactivation of CRTAP (*Crtap*^−/−^) and another with a knock-in of the G610C mutation of *Col1a2* (*Col1a2*^*tm*1.1*Mcbr*^). In both models, treatment with an antibody that neutralized the effect of TGFβ increased bone mass and bone structure and reduced bone turnover. Based on these data, fresolimumab, a monoclonal antibody which inhibits all three isoforms of TGFβ is currently being investigated in a phase I trial (NCT03064074) in adults with osteogenesis imperfecta.

## Cell Therapy

Bone marrow transplantation has been investigated as a possible means of treating severe OI. The first clinical study in this area was that of Horwitz and colleagues who performed bone marrow transplantation in three individuals with type III severe deforming OI (53). Two were aged 13 months and one 32 months. The donor marrow was obtained from unaffected HLA identical or single antigen mismatched siblings and infused intravenously without modifications. The recipients also had preconditioning with cytotoxic therapy and in one case, total body irradiation. This was an exploratory study with no control group. In order to determine whether the procedure was successful in engrafting cells of the osteoblast lineage to the recipients, the authors were able to culture osteoblasts from bone marrow about 3 months after the procedure in two patients and estimated that between 1.5 and 2% were of donor origin. Some changes in bone mass, linear growth and bone histology were observed post-transplant. In two patients the procedure was well-tolerated but in the third individual it was complicated by sepsis, pulmonary compromise and a hygroma.

The same group of investigators performed a further study in five children of a similar age. All had type III OI and were treated using a similar approach (54). In two of these individuals the researchers were unable to document donor osteoblast engraftment after the procedure and they were excluded from analysis. A further two individuals with type III OI of a similar age acted as controls. The authors reported an increase in growth velocity following the procedure which was not observed in the controls but this subsequently slowed. The treated individuals showed a gain in total body mineral content following the procedure but data were not reported for the control subjects.

Subsequently, the same group of researchers investigated the effects of mesenchymal stem cells (MSC) on growth and bone mineral content in a further six patients with type III OI aged between 3 and 5 years (55). In this case bone marrow was obtained from donors and then cultured *in vitro* with the aim of selecting MSC by their ability to adhere to the plastic surface of culture dishes. The adherent MSC were then transfected with retrovirus encoding marker genes; one was a neomycin resistance gene which was expressed; the other was a cassette for a neomycin resistance gene and a beta-galactosidase gene which were not expressed. The purpose of transfecting the MSC with these sequences was simply to provide a means by which the transplanted cells could be detected. The study showed that engraftment with the donor cells in bone, bone marrow and skin was variable but that the proportion of engrafted cells (estimated by PCR of target tissues) never exceeded 1%. Most of the treated individuals showed an increased growth velocity in the 6 months after transplant but in four cases this was not accompanied by an increase in bone mineral content. Adverse events included an urticarial rash in one treated patient and the development of antibodies to bovine serum in another.

It is difficult to interpret whether or not the treated individuals derived clinical benefit from the transplant procedure due to limitations in study design and the short term follow up of participants. Based on the information available however it seems unlikely that bone marrow transplantation or MSC transplantation has durable clinical benefits in severe OI.

## Gene Based Therapies

Correcting the genetic defects that cause OI would represent an attractive approach to treatment that could potentially be curative. This has been explored in the studies of Chamberlain et al. (56, 57) who used an Adeno-Associated Virus to target exon 1 of the *COLIA1* gene with the aim of replacing a deleterious missense mutation with a non-functioning allele using homologous recombination *in vitro*. The authors were able to demonstrate that it was possible to target cultured mesenchymal stem cells from affected patients using this approach and correct the abnormalities in collagen processing, stability and structure. In order for this approach to be used clinically, the next step would be to perform an autograft with the targeted cells but this has not been attempted.

Further gene-based approaches that could be explored in severe OI but which have not yet been attempted are to try and correct the genetic defect in stem cells or mesenchymal cells using CRISPR-Cas technology which is being trialed in Thalassaemia (NCT03655678) or to use anti-sense RNA or oligonucleotides to suppress expression of damaging mutant transcripts (58).

## Orthopedic Management of Osteogenesis Imperfecta

The aims of the orthopedic management are to maximize function and minimize pain for the patients affected. As mentioned previously, this is best done within a multidisciplinary team setting consisting of physiotherapists, orthotists, and rheumatologists or endocrinologists specializing in the medical management of OI. Orthopedic treatment begins in early life and remaining physically active is very important both for the strength of the bone and muscle as well as for the general well-being of the patient.

### Treatment of Fractures

Children with Osteogenesis Imperfecta unfortunately become very well-known to their local Accident and Emergency and Orthopedic Services due to recurrent fractures. A conservative approach in the orthopedic management of fractures in patients with OI is usually taken and fixation is rarely performed in the acute fracture setting. Splinting and casting is normally used and after a number of fractures the parents and patients themselves become very adept at applying their own cast and indeed will often do this at home reducing the need for hospital visits. The local policy in NHS Lothian is to use “soft cast” which is a pliable material which is kind to the soft tissues but provides enough stability to the extremity to allow the fracture to heal. Early mobilization is encouraged and if the child is ambulant prior to a lower limb fracture then weight bearing as soon as tolerated is the main aim as repetitive periods of non-weight bearing lead to muscle wastage, fatigue and potentially the loss of ability to independently ambulate. The prevalence of fractures in OI drops in adulthood but they continue to occur such that 25% of lifetime fractures are reported to occur in adulthood. Fractures in adulthood can be even more challenging due to the presence of deformity, previous surgery and/or metalwork and previous or ongoing bisphosphonate treatment (59).

### Orthopedic Management of Deformity

The most challenging aspect that OI patient's provide to the orthopedic surgeon is the management of their sometimes severe and multiplanar deformities. As already noted, the patient should be managed within a multidisciplinary setting and the medical management of the bone quality be optimized where possible. Functional gains are the main indication for deformity correction surgery and this has greater implications in the ambulant patient. The use of multiple long bone osteotomies secured with intramedullary rod fixation was described by Sofield in the 1950's and this principle continues to be used to the current time (60). This corrects all of the deformities in single long bone in one surgical event and is fixed with an intramedullary nail. The intramedullary fixation device is much less likely to give rise to a peri-prosthetic fracture compared to other fixation devices and is thus seen as the fixation method of choice in these patients. In children, telescopic rods such as the Fassier-Duval and the Sheffield telescopic intramedullary rod system have gained popularity. These have been shown to correct deformity, prevent fractures, and enhance function (61, 62). However, re-operation and complication rates are high. Delayed and non-union is often encountered and repeat surgery incorporating the use of bone graft is often required (63). Over recent times a more proactive approach has been taken to the upper limb in correcting deformities of the humerus and forearm (64, 65). This is due to the functional importance of this in wheelchair operation and the performance of independent self-care. However, again these are complex surgeries and post-operative problems are common. High rates of revision surgery are reported from most centers (66).

Deformity of the spine including scoliosis, kyphosis, spondylolisthesis, and base of skull problems are also commonly seen in OI patients, particularly adolescents. The mechanisms of scoliosis are incompletely understood but it is associated with low BMD and low BMI (67) The scoliosis is thought to be due in part to vertebral fractures coupled with abnormalities of the vertebral growth plates (68). The influence of medical treatment on the spine in OI is uncertain. Bisphosphonate therapy was associated with slowing of the rate of progression of scoliosis in a retrospective observational study of 316 children with OI but only found in a subgroup of patients with type IV OI that were treated before the age of 6 years (69). A prospective randomized trial would be required to determine if this apparent benefit is due to the intervention or confounding factors.

The orthopedic management of scoliosis is challenging and reduced bone quality makes spinal fusion difficult. Fusion performed before the curve becomes too severe (Cobb angle 45 degrees) is preferred and is known to prevent respiratory complications in these patients (70). Cranio-cervical junction abnormalities such as basilar invagination may also observed in OI and can results in hydrocephalus and brainstem problems. The input of experienced neurosurgeons in regard to this and treatment with cranio-cervical fusion can be required and comes with the same challenges as noted in extremity and spinal orthopedic surgery (71). Spondylolisthesis at the L5 level is found in up to 10% of patients with OI; these patients often have an increased lumbar lordosis predisposing them to this condition and again careful consideration of this should be given when assessing an OI patient with any lower limb neurological symptoms (72).

### The Osteogenesis Imperfecta Patient in the Operating Theater

Patients with OI who do need to come to theater provide additional challenges. Great care must be taken during the transfer of such patients on and off the operating table to prevent iatrogenic fracture. Careful pre-assessment by the anesthetic team should be carried out and precautions taken in airway management both due to the fact that the shape of the airway is often abnormal and the risk of cervical spine fracture during intubation. Lung function and bleeding should be taken into particular consideration during surgical correction of spinal deformity in OI (73).

## Author Contributions

The manuscript was jointly written by SR and MG.

### Conflict of Interest

The authors declare that the research was conducted in the absence of any commercial or financial relationships that could be construed as a potential conflict of interest.
